# Detection and clinical significance of circulating tumor cells in colorectal cancer

**DOI:** 10.1186/s40364-021-00326-4

**Published:** 2021-11-19

**Authors:** Miao Jiang, Shuiling Jin, Jinming Han, Tong Li, Jianxiang Shi, Qian Zhong, Wen Li, Wenxue Tang, Qinqin Huang, Hong Zong

**Affiliations:** 1 Department of Oncology, the First Affiliated Hospital of Zhengzhou University, NO.1 Eastern Jianshe Road, Zhengzhou, 450052 Henan China; 2 BGI College, Zhengzhou University, 40 Daxue Road, Zhengzhou, 450052 Henan China; 3 Precision Medicine Center, Henan Institute of Medical and Pharmaceutical Sciences, Zhengzhou University, 40 Daxue Road, Zhengzhou, 450052 China; 4 Departments of Otolaryngology, The Second Affiliated Hospital of Zhengzhou University, Zhengzhou, 450000 Henan China; 5 Academy of medical science, Zhengzhou University, Zhengzhou, 450052 Henan China

**Keywords:** Colorectal Cancer, Circulating tumor cell, Cellular heterogeneity, Precision medicine, Separation

## Abstract

Histopathological examination (biopsy) is the “gold standard” for the diagnosis of colorectal cancer (CRC). However, biopsy is an invasive method, and due to the temporal and spatial heterogeneity of the tumor, a single biopsy cannot reveal the comprehensive biological characteristics and dynamic changes of the tumor. Therefore, there is a need for new biomarkers to improve CRC diagnosis and to monitor and treat CRC patients. Numerous studies have shown that “liquid biopsy” is a promising minimally invasive method for early CRC detection. A liquid biopsy mainly samples circulating tumor cells (CTCs), circulating tumor DNA (ctDNA), microRNA (miRNA) and extracellular vesicles (EVs). CTCs are malignant cells that are shed from the primary tumors and/or metastases into the peripheral circulation. CTCs carry information on both primary tumors and metastases that can reflect dynamic changes in tumors in a timely manner. As a promising biomarker, CTCs can be used for early disease detection, treatment response and disease progression evaluation, disease mechanism elucidation, and therapeutic target identification for drug development. This review will discuss currently available technologies for plasma CTC isolation and detection, their utility in the management of CRC patients and future research directions.

## Introduction

Colorectal cancer (CRC) is the fourth deadliest cancer in the word, with almost one million deaths annually [1]. In recent years, its morbidity and mortality have increased due to delayed diagnosis and limited treatment options [2]. Early detection and precision treatment are keys to improving the prognosis of CRC patients [3]. In the past, the definite diagnosis of CRC mainly relied on biopsy-based techniques [4]. One single static biopsy specimen cannot reflect the characteristics of the tumor in real time. Therefore, there is an urgent need for a safer and more real-time method to obtain comprehensive and dynamic information that can reflect the development and treatment response of cancer to supplement or replace solid tissue biopsies.

“Liquid biopsy” has attracted widespread attention from researchers [5–7]. Liquid biopsy refers to the analysis of biomarkers including circulating tumor cells (CTCs), circulating tumor DNA (ctDNA), microRNA (miRNA) and extracellular vesicles (EVs) in peripheral blood or other body fluids [8] This method can overcome the limitations of solid biopsy and reflect the heterogeneous information of the tumor in real time.

CTCs are epithelial cancer cells that have the ability to move, migrate and invade blood vessels after epithelial-mesenchymal transition (EMT) and are considered the main cause of tumor metastasis in vivo [9, 10]. Upon reaching an appropriate niche, CTCs undergo mesenchymal-epithelial transition (MET), reacquire stem cell properties and form a new metastatic site [11]. Unlike other cancer biomarkers, CTCs are live tumor cells that carry molecular and biological information about the tumor as a whole, support single cell analysis, and directly reflect the ongoing changes in tumors at all stages [12].

Studies of different cohorts have shown that the quantity of CTCs is valuable for assessing the prognosis of CRC [13, 14]. CTCs also show application value in early diagnosis and monitoring the dynamic changes [15, 16]. The phenotypic and molecular characteristics of CTCs can help reveal the mechanism of pathogenesis and metastasis of CRC and identify specific mutations in target genes [17, 18]. The value of CTCs in CRC is gradually being confirmed; however, the rarity and heterogeneity of CTCs as well as the development of detection and analysis technologies have limited the widespread acceptance and application of CTCs as a new biomarker. In recent years, a variety of new platforms that can detect different subtypes of CTCs with high sensitivity and specificity have been developed. Researchers are continuing to verify the correlations between CTC characteristics and other clinical characteristics. This review will provide an overview of current and emerging techniques of CTC isolation and detection and discuss their potential clinical value.

## CTC enrichment and identification

**Fig. 1:**
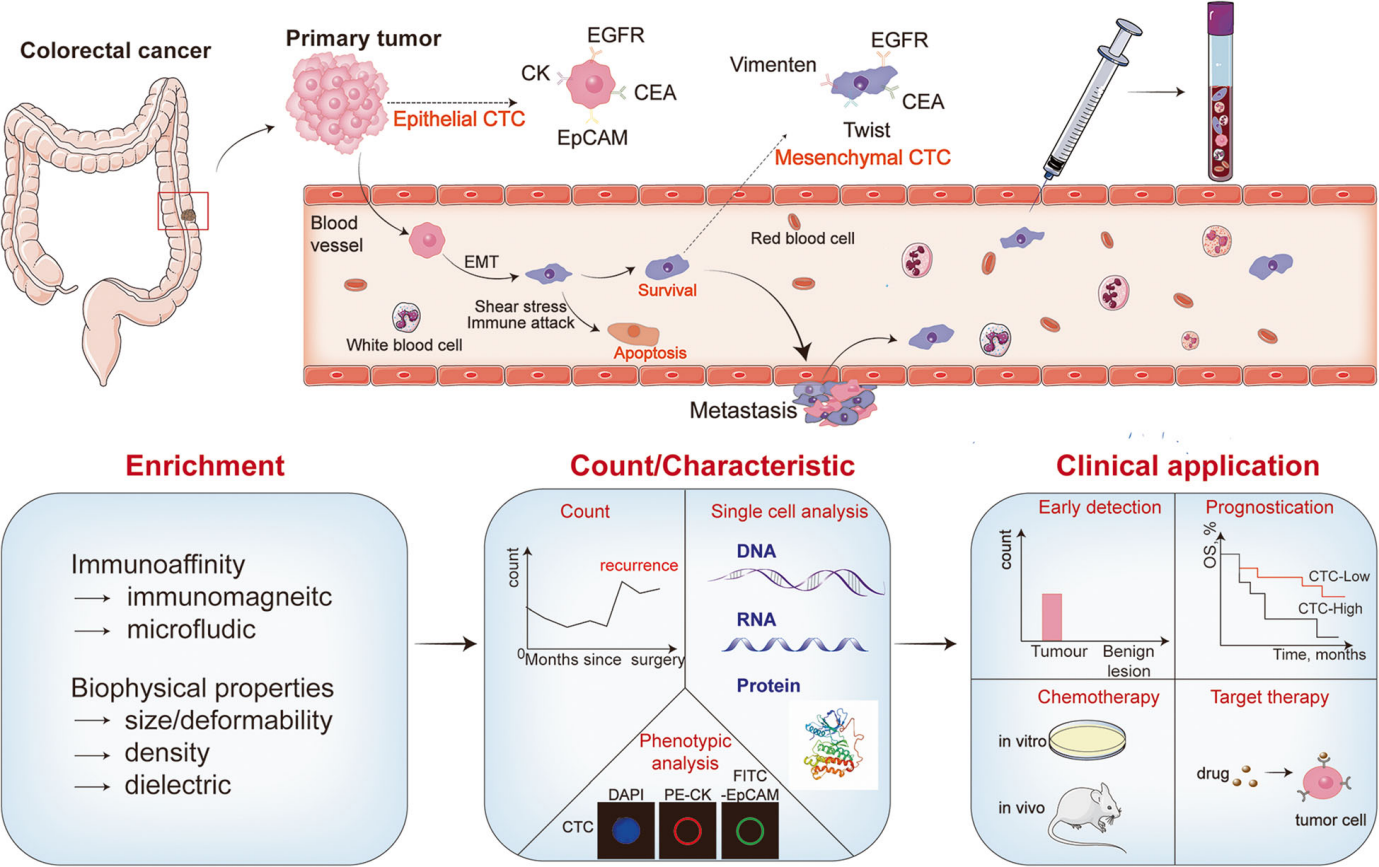
The roles of CTC in tumor metastasis and the current applications of CTC technologies. Tumor cells translocate from the primary tumor and invade into the bloodstream to become CTCs. Most CTCs affected by microenvironment and undergo apoptosis. Some CTCs undergo phenotypic changes after EMT and finally forming metastases. CTCs are preliminary enriched from whole blood sample via different enrichment techniques. Different detection technologies such as CTC count, phenotypic analysis and single cell analysis can help with early detection, prognostication, chemotherapy, target therapy of patients

CTCs exist before tumor lesions can be detected by medical imaging examination [19]. Patients with advanced tumors will typically have only 1 CTC out of 1 × 10^6^ neutrophils in 1 mL of peripheral blood, and the number of CTCs in early-stage patients is even lower [20]. In addition, CTCs are susceptible to apoptosis and inherent fragility. When detached from the original focus, they are prone to undergoing phenotypic changes, and only a few cells can survive [21] (Fig. 1). Liquid biopsy allows repeated sampling and has the potential to assess tumor heterogeneity and the clonal diversity of drug resistance. Such information is of great significance for accurate drug administration and improving patient prognosis. Several systems have emerged to improve the detection efficiency of CTCs, which can be broadly classified into two categories: immunoaffinity-based enrichment and biophysical property-based enrichment (Table 1).

**Table 1:** CTC Detection Technologies

Principle	Material/Device	Characteristic	Target	Tumor Type	Sensitivity%	Specificity%	Viability%	Ref
**Immunoaffinity**	Immunomagnetic	Immunonanocomposites equipped with fluorescence and magnetic properties (ZnS:Mn2+ quantum dots and Fe3O4/SiO2), which achieved capture and identification of CTCs simultaneously.	EpCAM	CRC/BC	90.8	–	–	[23]
	Nanofibers	Poly ethylene oxide(PEO) was blended into nylon-6 through electrospinning to produce a fibrous mat-based CTCs detection method. High cell-substrate affinity and low leukocyte adsorption.	EpCAM	CRC	66.7–70.6	> 99	–	[24]
	bio-microchip	ZnO nanowire coated polydimethylsiloxane (PDMS) pillar microchips with a gear structure. More binding sites and rough surface.	EpCAM	CRC/BC	91.11 ± 5.53	–	96	[25]
	Immunomagnetic	Tumor-targeting molecule FA and magnetic nanoparticles (MNPs) coated red blood cells. Natural cell membrane avoided absorption by leukocytes.	FR	BC	> 90	> 75	> 90	[26]
	Nanovesicles	FA and fluorescein Cy5 coated erythrocyte-derived nanovesicles. Achieving Tumor targeting and fluorescence imaging simultaneously.	FR	CRC/BC	> 95	> 90	–	[27]
	Fluorescent probe	FA functionalized terbium-doped dendritic fibrous nanosilica fluorescence spectrometry. Fluorescence microscopy and flow cytometry were utilized to verify the uptake of the fluorescent probe with FR-positive tumor cells.	FR	CRC	85	–	> 80	[28]
	Immunomagnetic	rVAR2 protein coated micromagnetic beads. rVAR2 binding is unaffected by phenotypic plasticity.	ofCS	HCC/LC/PAAD/PC	86.0–91.9	–	–	[30]
	Immunomagnetic	rVAR2 protein coated micromagnetic beads.	ofCS	GBMLGG	54–75	–	–	[31]
	Microfluidic	Membrane protein O-glycan sialyl-Tn (STn) glycan-affinity microfluidic devices.	sTn	CRC	22	> 99.5	–	[36]
	Immunomagnetic	EpCAM incombination with EGFR and HER3 based enrichment can significantly improve the detection rate of CTCs.	EpCAM/EGFR/HER3	NSCLC	66.7	–	–	[37]
	Immuno-microbeads	Anti-EpCAM and anti-CD146 antibodies functionalized gelatin nanoparticle-coated silicon microbeads (SiO2@Gel MBs). SiO2@Gel MBs have large size and high density. The gelatin coating allows the release of captured CTCs with low damage and high efficiency.	EpCAM/CD146	BC/CRC	> 80	> 85	92.5	[38]
	Immunomagnetic	FA and anti-EpCAM antibody functionalized magnetic hyaluronan capsules.	EpCAM/FR	BC	> 88	95	–	[39]
	Microfluidic	A platform integrated of dendrimer-mediated multivalent binding, a mixture of antibodies and biomimetic cell rolling.	EPCAM/EGFR/CA9/c-Met	RCC	80	–	–	[40]
	Magnetic dynamic microbiointerface	A saccharide-sensitive biomimetic microplatform, which can mimick the dynamic properties of natural excellular matrix. Good biocompatibility, high capture efficiency, excellent cell selectivity.	mussel-inspired cancer cell-targeting peptide	BC	85	–	–	[42]
	AuNPs Electrochemical	Gold nanoparticle-based multipedal DNA walker. TCEP-mediated electrochemical amplification is used to further enhance the electrochemical signal. High sensitivity and anti-interference ability.	DNA probes	ALL	> 96.3	–	–	[43]
**Biophysical Properties**	Microfluidic	A spiral microchannel with inherent centrifugal force. Taking advantage of two phenomena: Dean migration and inertial focusing. Only the larger CTC undergo inertial focusing and be collected.		LC	100	10	> 98	[52]
	Microfiltration	A 3D palladium filter with an 8 mm-sized pore and a 30 mm-sized pocket. A device driven by gravity flow.		BC/GC/CRC/PAAD	> 85	–	–	[53]
	Microfiltration	A high-density microporous (HDM) chip. High sensitivity and specificity, simplicity, and low cost, enables subsequent single cell molecular characterization and analysis.		CRC	65.8	96	–	[54]
	Density gradient centrifugation	The unique separation tube and collector of AccuCyte system enable the complete separation of the buffy coat layer of white blood cells and platelets.		BC/PC/CRC	90.5	81	–	[57]
	Microfluidic	Consist of deterministic lateral displacement (DLD) isolating structure, an automatic purifying device with CD45-labeled immunomagnetic beads and a rat-tail collagen coated capturing platform.		LC	90	50	> 90	[58]
	Microfluidic	A sequential ensemble decision aliquot ranking (eDAR) chip with two sorting nodes. CTCs are detected by laser-induced fluorescence(PE-EpCAM), then the CTC-detection signals activate a solenoid and change the pressure of flow to achieve sorting at two junctions.		BC	> 90	70	–	[61]

### Immunoaffinity

Immunoaffinity-based CTC enrichment techniques, which use molecular probes such as antibodies, peptide chains, and aptamers, are the most common category. The most widely used immunomagnetic separation methods are positive enrichment based on epithelial cell adhesion molecule (EpCAM) and negative enrichment based on CD45. The most representative system is the CellSearch system, which is the first and only system to meet the standard of the US Food and Drug Administration (FDA). The CellSearch system utilizes ferrofluid beads coated with EpCAM antibodies and then identifies cells using 4′,6-diamidino-2-phenylindole (DAPI)-based cell nuclear staining, CD45-allophycocyanin-specific leukocyte negative selection and cytokeratin 8,18,19-phycoerythrin-specific epithelial cell positive selection to creat an objective indicator (EpCAM+, DAPI+, CD45-, cytokeratin+) of CTC counts. The CellSearch system has been approved for the diagnosis and prognosis of patients with CRC, breast cancer and prostate cancer [22].

**Fig. 2:**
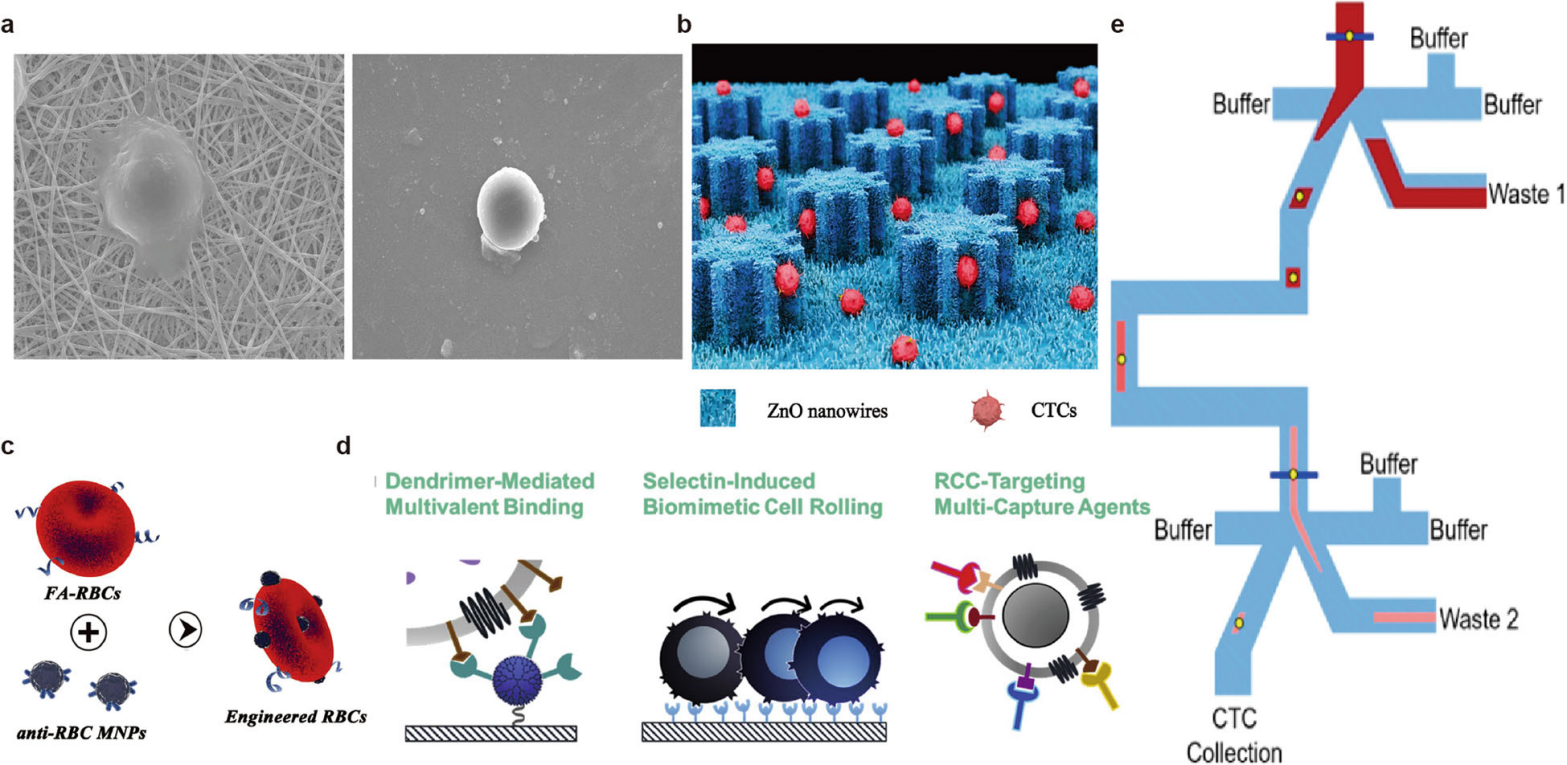
Strategies for CTC enrichment. **a** A fiber mat of electrospun nylon-6/PEO fibre for CTCs capture; **b** A ZnO nanowire coated polydimethylsiloxane pillar substrate with a gear structure; **c** Tumor-targeting molecule folic acid (FA) and magnetic nanoparticles (MNPs) coated engineered red blood cells (RBCs); **d** A microfluidic device integrated of dendrimer-mediated multivalent binding, a mixture of antibodies, and biomimetic cell rolling; **e** An ensemble-decision aliquot ranking (eDAR) microfluidic device using sequential sorting and flow stretching. **a** Copyright Springer Nature 2018. Reproduced with permission from reference [24]; **b** Copyright RSC Publishing 2020. Reproduced with permission from reference [25]; **c** Copyright RSC Publishing 2018. Reproduced with permission from reference [26]; **d** Copyright Elsevier 2020. Reproduced with permission from reference [40]; **e** Copyright American Chemical Society 2019. Reproduced with permission from reference [61]

EpCAM, which is representative of the markers used in positive enrichment techniques, is epithelial-specific and is strongly expressed in most cancers. In recent years, a variety of CTC enrichment technologies based on EpCAM have emerged. Cui et al. engineered a fluorescent and magnetic immunonanocomposite (ZnS:Mn^2+^ quantum dots (QDs) and Fe_3_O_4_/SiO_2_) modified with an EpCAM antibody for fluorescence identification of CTCs by the fluorescence emitted by ZnS:Mn^2+^ QDs during the capture of CTCs. This method omitted the complex steps of traditional three-color fluorescence identification and efficiently reduced cell damage and loss [23. Lee et al. engineered an EpCAM antibody-modified binary-blend fiber [24] (Fig. 2a). By blending polyethylene oxide (PEO) into nylon-6 by electrospinning, nonspecific binding was reduced while providing a rough nanosubstrate, which facilitated high cell-substrate affinity and low leukocyte adsorption. The blood samples of 23 CRC patients were analyzed, and the results were compared with those of colonoscopic biopsy and the IsoFlux system. They concluded that the new method could be used for the diagnosis of CRC via CTC status. In addition to the properties of the material itself, the surface properties of the material can also be optimized. Cui et al. constructed novel EpCAM antibody-functionalized ZnO nanowire-integrated biomicrochips [25] (Fig. 2b). ZnO nanowire arrays provided a rough surface for gear structure polydimethylsiloxane(G-PDMS), while the gear structure provided more binding sites for antibodies and target cancer cells. The microchip achieved a high capture rate (91.11% ± 5.53%) and release rate (> 90%) of CTCs, and the high sensitivity of ZnO nanowires to pH enabled the captured CTCs to be released quickly, efficiently, and with minimal damage under weakly acidic conditions.

Metastasis is the major challenge in cancer management. Many studies have suggested that primary tumor cells need to undergo EMT before invading blood vessels and gaining metastatic ability. These cells experience different degrees of EMT and gain several different subtypes, including the epithelial type, mesenchymal type or mixed type, and the expression of EpCAM on the surface of CTCs is downregulated to varying degrees, making it difficult for these heterogeneous cells to be enriched by EpCAM-dependent CTC capture technologies. These tumor cell subsets are of great significance for studying the mechanism of cancer transmission and metastasis. Therefore, researchers have improved many aspects of CTC capture techniques, such as antibodies, biological probes, and the source of blood samples.

Optimizing antibody application strategies, such as searching for different CTC-specific biomarkers, is an effective approach to improve CTC capture efficiency. To meet the demand of rapid division, folate receptors (FRs) are overexpressed on the surface of most tumor cells to absorb folic acid (FA) to promote the biosynthesis of DNA and RNA. Zhu et al. developed FA-coated and magnetic nanoparticle-coated engineered homogeneous red blood cells and obtained a high capture rate (> 90%) and survival rate (> 90%) in artificial whole blood samples; in addition, the purity (> 75%) was much higher than that of EpCAM-based immunomagnetic separation technology (20%) [26] (Fig. 2c). Complexes in physiological environments can cover the surface of the magnetic beads, affecting their binding to tumor cells. Moreover, nonspecific binding with white blood cells is high, leading to a decline in purity. The immune escape mechanism of natural cells greatly reduces nonspecific binding, showing their potential as highly specific CTC capture materials. Chen et al. engineered an FA modified erythrocyte-derived nanovesicle as a tumor-targeting molecules [27]. They achieved a high capture efficiency (> 90%) in whole blood, while magnetic-activated cell sorting (MACS) reached only 50%. Jafar et al. developed FA-functionalized terbium-doped dendritic fibrous nanosilica and achieved a lower limit of detection of 500 cells/ml for HT29 cancer cells by spectrofluorimetric cytosensing [28]. Oncofetal chondroitin sulfate is specifically expressed in the placenta and in almost all types of tumor cells, indicating that it may be an ideal target. In 2015, a smaller recombinant VAR2CSA protein encompassing ID1-ID2a (rVAR2) was shown to specifically bind to 95% (106/111) of different phenotypes of human cancer cells with little binding to normal tissue cells [29]. rVAR2 has great potential for CTC-specific capture and targeted drug therapy. In 2018, the same group presented a CTC isolation method based on rVAR2-modified micrometer-scale magnetic beads and the IsoFlux System [30]. They confirmed the specificity of rVAR2 for tumor cells, which was independent of cell phenotypes [31]. rVAR2 could potentially be used to target different types of cancer cells and guide precision medicine [32–34]. In 2020, they optimized several parameters of this technology [35], including the size and concentration of the magnetic beads and the recombinant forms and concentration of rVAR2. Compared with the widely used direct CTC capture approach, the indirect capture approach of preincubating cells and antibodies was more effective for rVAR2-based cancer cell retrieval under conditions of low target expression. Their findings may serve as an inspiration for the development and optimization of other bead-based CTC capture technologies. Membrane protein O-glycan sialyl-Tn (STn) antigen is overexpressed in advanced digestive tract tumors such as CRC and gastric cancer but not in normal blood cells and is often associated with tumor metastasis and poor prognosis. Manuel et al. proposed that for the same CRC patient, STn^+^ CTC counts were significantly higher than EpCAM^+^ CTCs counts, and they constructed a glycan affinity-based microfluidic device that improved the separation efficiency of CTCs in the peripheral blood of CRC and bladder cancer patients [36].

However, the application of new markers also faces the obstacle of the heterogeneity of ligand expression levels. Exploiting synergism between antibodies may improve the sensitivity of CTC detection technologies. In a cohort of 45 non-small cell lung cancer (NSCLC) patients [37], the positive rate of CellSearch was only 33%, and the enrichment based on EGFR/HER3 was 37.8%. When both antibodies were combined, the positive rate (66.7%) increased significantly. Huang et al. functionalized gelatin nanoparticle-coated silicon microbeads (SiO_2_@Gel MBs) with anti-EpCAM and anti-CD146 antibodies to enhance the capture rate of mesenchymal CTCs with low EpCAM expression [38]. Compared with the traditional EpCAM-based method, higher capture efficiency (> 80%) and higher purity (> 85%) were obtained. Ma et al. constructed multitargeting magnetic capsules (TMCs) with an anti-EpCAM antibody and FA and captured and isolated more than 88% of tumor cells in 15 min of incubation, with high specificity [39]. Jiyoon et al. developed a novel capture platform utilizing four antibodies against renal cell carcinoma (RCC) CTCs, including EpCAM, carbonic anhydrase IX (CA9), epidermal growth factor receptor (EGFR) and hepatocyte growth factor receptor (c-Met). This platform increased the capture rate of RCC cells to up to 80% and was significantly superior to other capture surfaces functionalized with a single antibody(≤60%) [40] (Fig. 2d). However, not all strategies can improve capture performance. Tim N et al. used two methods to capture CTCs: the standard EpCAM CellSearch kit (unicapture) and the combined ferrofluid capture of EpCAM plus HER2-, EGFR- and MUC-1-specific antibodies (quadcapture). However, the quadcapture ferrofluid reagent failed to significantly improve the CTC capture efficacy [41]. It is necessary to explore and verify different antibody combination strategies.

In addition to specific antibodies, a variety of molecular probes can be used to target tumor cells. Tian et al. constructed a magnetic dynamic microbiointerface with a biofeedback mechanism that mimicked the dynamic properties of the natural extracellular matrix (ECM) and reversible ligand−receptor interactions [42]. This saccharide-sensitive biomimetic microplatform could release CTCs via a molecular exchange mechanism with low damage and achieved high cell capture (85%) and release (93%) efficiencies. Peng et al. developed a gold nanoparticle-based multiple DNA walker for the ultrasensitive detection of CTCs for the first time (capture rate > 96.3%) [43]. By modifying a number of walker strands on gold nanoparticles (AuNPs), the integrated aptamer sequence can specifically bind to the transmembrane receptor protein of CTCs. During DNA walking, several tracks hybridized with the electrode simultaneously for subsequent nicking endonuclease-catalyzed cleavage. Then, TCEP-mediated electrochemical amplification was employed to obtain an enhanced electrochemical signal. The lower limit of detection was 1 cell/ml. The outstanding resolution of CTCs in blood samples suggests their potential application prospects in early diagnosis of cancer.

Most in vitro CTC enrichment technologies rely on small sample volumes (5–10 ml), and researchers strive to improve the sensitivity. Due to the scarcity and uneven distribution of CTCs, the usual sources and volume of peripheral blood samples limit the improvement of the technology. In vivo CTC detection technology has aroused the interest of researchers. This kind of technology can continuously process large volumes of blood to simultaneously obtain more CTCs and enable dynamic monitoring. At present, there are two main types of in vivo detection technologies. One representative technology is CellCollector (GILUPI CellCollector, GILUPI, Potsdam, Germany) [44]. A wire modified by an EpCAM antibody is placed into the vein of the arm for 30 min, and then the tumor cells on the steel wire are identified under a fluorescence microscope for early cancer diagnosis. However, it can only be used for CTC counting, and no further analysis can be carried out. Moreover, the specific blood volume varies between patients and at different time points for the same patient, which makes it difficult to further evaluate characteristics such as the change in CTC count during treatment. Another kind of method is in vivo imaging flow cytometry [45]. By visualizing of tumor cells and blood vessels in advance, the interactions between cells circulating in the blood can be directly observed. This technique has great potential for observing the interactions among targeted drugs, immune cells and tumor cells. However, CTCs cannot be directly obtained for downstream analysis, and this kind of method may affect the physiological state of the patient. Tang et al. constructed an extracorporeal circulation system based on an in vivo microfluidic chip detection system (IV-chip-system) [46]. Venous blood is continuously drained into the extracorporeal circulation system to detect and monitor CTCs, and then the blood is returned back to the body directly. This method enables the analysis of a large amount of blood with low damage to the body. At present, it has only been tested in mouse models, and more research is needed to evaluate the feasibility of this technique in the human body.

The identification of CTCs after enrichment still depends on epithelial biomarkers. The damage and loss of CTCs in the complex fluorescence labeling process can be avoided by developing a post-enrichment identification method. Li et al. constructed a hydrogen peroxide-responsive nanoprobe [47]. Tumor cells have an exuberant metabolism and high levels of endogenous hydrogen peroxide, and the probe can quickly respond to high levels of intracellular hydrogen peroxide and emit fluorescence signals. Besides increasing the volume of blood samples, the source of blood samples can be expanded to increase the number of CTCs detected. Researchers found that more CTCs were detected in tumor-drained venous blood or mesenteric-drained venous blood than in peripheral blood [48–50]. CTCs were detected before the liver first-pass effect to reduce the negative effect on the number of CTCs in peripheral blood. Compared with the conventional detection of peripheral blood, the detection rate and capture rate were higher.

### Biophysical properties

Biophysical property-based enrichment technology mainly exploits the differences in size, deformability, density and dielectric properties between CTCs and blood cells, which eliminates need to address the heterogeneity of cell surface antigen expression. In addition, the isolated CTCs are more suitable for downstream analysis without antibody labeling or special treatment [51]. CTCs of various phenotypes are captured almost indiscriminately. The comprehensive analysis of different phenotypes of CTCs can facilitate the determination of the value of CTCs in guiding treatment and predicting prognosis.

The size-based devices isolate CTCs mainly utilize the physical and mechanical differences between CTCs (10–20 μm) and other blood components (red blood cells, 8 μm; leukocytes, 7–12 μm; better deformability). Since there is some degree of overlap in the size of CTCs and leukocytes, it’s crucial to choose an appropriate threshold. A size of 8 μm is usually considered the appropriate cutoff value. Taking advantage of the size difference between CTCs and hematologic cells, Han et al. constructed a spiral microchannel with a recovery rate of more than 85% of a breast cancer cell line (MCF-7) using centrifugal force [52]. Under the influence of Dean drug forces, all the cells migrate from the outer channel to the inner channel along the Dean vortex. The smaller cells will eventually return to the outer wall and be discarded, while the larger cells will experience additional strong inertial lift forces and focus along the inner wall to be collected. Akiko et al. developed a 3-dimensional (3D) palladium filter driven by gravity flow that can recover CTCs from whole blood with a recovery rate of more than 85% [53]. Bo Young Oh et al. used a high-density microporous (HDM) chip to detect CTCs in 76 CRC patients [54]. Whole blood samples were pretreated and filtered with the HDM chip, and the positive rate of CTCs was 65.8%. Anil et al. constructed an electrical double layer (EDL)-gated AlGaN/GaN high electron mobility transistor (HEMT) biosensor array that utilizes the response to cell transmembrane potential to detect and count tumor cells and can also be used to monitor the dynamic transformation of fine membrane potential [55]. Parham et al. utilized the specific differential impedance curve generated when CTCs are deformed through an 8 μm channel. In addition to identification and counting, the accuracy of identifying the origin of cancer cells was more than 90% [56].

The density of CTCs is intermediated between that of plasma and red blood cells but within the range of leukocyte density. By taking advantages of the different sedimentation coefficient in the density medium (such as Ficoll-Paque medium) and density-based gradient centrifugation, cells of different densities are distributed in layers in the separation solution to realize the separation of CTCs. For example, the principle of the AccuCyte system is to use the specific density of CTCs, but unlike other density-based separation methods, its unique separation tube and collector completely separate the buffy coat layer of white blood cells and platelets, avoiding the loss of a large number of CTCs [57]. After separation, CTCs are identified and characterized by a CyteFinder system. The average recovery rate for suspended tumor cells was 90.5%. Verification in a clinical cohort of CRC revealed that the CTC counting ability of the system was better than that of CellSearch. However, there is some overlap of physical properties between leukocytes and CTCs, which will lead to poor specificity and the loss of some CTCs.

CTC enrichment techniques based on immune affinity and biophysical properties have their own advantages and limitations. The emergence of integrated microfluidic devices combines the advantages of both and improves the sensitivity and specificity of monitoring and detection. In addition, the enrichment, identification and analysis of CTCs can be realized on one platform, which saves time and reduces the damage and loss of CTCs during the transfer process. Jiang et al. constructed an integrated microfluidic device consisting of a deterministic lateral displacement (DLD)-isolating structure, an automatic purifying device with CD45-labeled immunomagnetic beads and a rat tail collagen-coated capturing platform, which can capture cancer cells with high throughput [58]. Taking advantage of fluid characteristics, negative enrichment of multistep purification achieved a 90% capture rate, 50% purity and over 90% survival rate. The positive rate in clinical patients was 83.3%. There was no statistical difference in the capture rate between this system and CellSearch, but this system has the advantages of blood volume, time and cost. The same research group then optimized the staining and separation steps of the device and the obtained CTCs could be analyzed at single­cell resolution [59]. They detected six gene mutations, indicating that this device can effectively obtain CTCs with little damage to their activity and can be used for downstream analysis. Eleanor S et al. designed a sequential ensemble decision aliquot ranking (eDAR) chip with two sorting nodes and realized the capture of CTCs with high purity relative to white blood cells in whole blood by making use of fluid characteristics. In previous studies, they proved that the eDAR platform could capture both EPCAM^high^ and EPCAM^low^ CTCs simultaneously, and the capture efficiency (> 90%) was relatively high [60]. After further improvements, cells could also be collected and transferred out of the chip, and one single CTC could be collected in a porous plate. The average number of contaminating leukocytes in each well was less than one, which enabled eDAR to realize the downstream analysis of a single CTC [61] (Fig. 2e). Chen et al. constructed an EpCAM antibody-functionalized microfluidic device by using 3D printing technology [62]. The interaction between CTCs and antibodies in the microfluidic channel was effectively improved by optimizing parameters such as intracavity surface area and fluid flow. Both EPCAM^+^ cells and EPCAM^−^ cells were successfully captured.

## Overview of the clinical application value of CTCs

CTCs entering circulation are heterogeneous groups from different tumor lesions and their phenotypic and molecular characteristics differ depending on the microenvironment and treatment. The detection and analysis of CTCs is a new approach for studying the heterogeneity of systemic tumors and promoting the development of precision medicine.  The merts and demerts of CTC-based methods, clinical methods and other biomarkers were summarized in (Table 2).

**Table 2:** Merits and Demerits of CTC-based methods, clinical methods and other biomarkers

Methods		Merits	Demerits
**CTCs**	Immunoaffinity-based	Capture cells of specific phenotypes with relatively high specificity, high purity; Overcome the heterogeneity of physical characteristic	Lack of broad-spectrum specific biomarkers; Affect cell viability, downstream phenotype identification and molecular analysis; High cost, low throughput
	Biophysical Properties-based	Good cell integrity and viability; Not restricted by cell surface markers; Low cost, high throughput	Low specificity and purity; Significant difference in cell size lead to the omission of small CTTs
	Commonality	Low-risk, Low-invasive method for continuous sample acquisition; Real-time (earlydiagnosis, predict metastasis or recurrence, monitor treatment response); Live cells, morphological and molecular characterization (precision medicine); In vitro culture, drug resistance and drug sensitivity cell experiments	Targeted cells are scarce; Technical limitations (sensitivity and specificity); Lack of uniform standards (cut-off value, detection time, etc.) Heterogeneity; Lack of large-scale prospective trials
**Clinical Methods**	Medical imaging	Mature, standardized; Non-invasive, dynamically monitor the morphological changes of lesions;	Insufficient sensitivity; Not in-time; The nature of some lesions is not clear
	Pathological	Mature, standardized; High accuracy; Immunohistochemical staining, genetic testing, in vitro cell culture, etc.	Invasive injury (local reaction, infection, dissemination and metastasis, etc.); Some patients cannot undergo tissue biopsy (location, size, general condition, etc.); Heterogeneity (time and space)
	Tumor biomarkers	Easy access to samples; Dynamic monitoring of tumor changes and treatment response	High false positive and false negative rate; Poor specificity
**Other biomarkers**	ctDNA	Relatively mature technologies (compared with CTCs); Short half-life time; More sensitive to tumor status; Comprehensive molecular information of tumor	DNA fragments from necrotic or apoptotic cells, cannot represent living tumor cells; Only provide genetic information; Low gene mutation abundance, poor sensitivity
	Extracellular Vesicles	Large quantity, easy to enrich (compared with CTCs); Extracellular vesicles can prevent internal nucleic acid substances from being degraded, high stability	Immature technologies (isolation, purification, enrichment of internal specific markers, etc.)
	microRNA	Closely related to cell metabolism under physiological and pathological conditions	Immature technologies, few related research

### CTC quantification

Evaluating the clinical and histopathological features of patients has significantly enhanced prognosis and treatment decisions for several cancer subtypes, but there is still much space for improvement. The quantification of serum prostate-specific antigen (PSA) has always been an important indicator of drug efficacy and prognosis in prostate cancer. A large docetaxel-based prospective cohort study of 212 prostate cancer patients found that adding day 0 CTC counts to baseline PSA and other covariates improved the survival prediction accuracy from 8 to 10%. After 3 weeks of treatment, the median overall survival (OS) of patients with increased CTC counts was more reliable than the PSA level for patient prognosis [63]. Satelli et al. found a significant correlation between CTC count and castration resistance in a cohort of 48 metastatic prostate cancer patients, whereas no significant correlation with the quantitative change in PSA was observed [64]. Transcriptional activity of CTCs collected before treatment in patients with different stages of prostate cancer was quantified digitally and patients with high CTC scores had worse responses to treatment and were prone to metastasis [65].

Similarly, in a meta-analysis of the data of 1944 patients with breast cancer, the researchers established a clinicopathological predictive model that includes CTC counts. The Likelihood ratio (LR) χ2 statistical analysis showed that this approach improved the accuracy of prognosis, whereas serum tumor markers (CEA and CA15–3) did not improve prognostic value [66]. An analysis of data from a large cohort of early breast cancer patients showed that patients who received radiotherapy after breast-conserving surgery had significantly longer local recurrence-free survival, disease-free survival (DFS), and OS than patients with CTCs detected before adjuvant therapy. This study suggested for the first time that CTC status can act as a predictive marker of local treatment benefits in patients with early breast cancer [67]. A randomized clinical trial (BRE12–158) analyzed the correlation between postoperative CTC status and follow-up prognosis in 123 patients with early triple-negative breast cancer. With distant disease-free survival (DDFS), DFS and OS as the primary outcome measures, the prognosis of CTC positive patients was significantly worse [68]. However, the above studies were limited by the CTC detection technology, follow-up time, patients’ information and other factors, and there remains an unmet need for prospective clinical trials to verify the value of CTCS. It is well known that the EMT process is closely related to metastasis. The proportion of mesenchymal CTCs combined with the total number of CTCs can provide more prognostic information. Guan et al. analyzed patients with metastatic breast cancer (MBC) and determined two thresholds: a total CTC count ≥10/5 ml and proportion of mesenchymal CTCs > 10.7%. The median progression-free survival (PFS) of those who meet the above conditions was significantly shorter [69]. CTC counts may serve as an early indicator for selecting treatment options. For hormone receptor-positive (HR+) MBC patients treated with letrozole plus bevacizumab (Let+Bev) or letrozole alone as a first-line treatment regimen, only patients who were baseline CTC positive showed a potential OS benefit from the Let+Bev regime [70]. If confirmed, CTCs may be an effective marker for predicting benefits in HR+ MBC patients treated with Bev or other targeted drugs.

The predictive value of CTCs in NSCLC has not been officially confirmed. However, according to current information, its potential as a predictive factor cannot be ignored. Chemi et al. detected CTCs in pulmonary vein blood collected during surgery (CellSearch, positive rate was 48%). The comparision of the genomic profiles of CTCs, primary lesions and metastases suggested that CTCs spread to the pulmonary vein during surgery and form metastatic foci; thus, CTCs derived from the pulmonary vein during surgery can serve as an independent predictor of recurrence [71]. Pulmonary venous blood can only be collected once, which limits the ability of CTCs to dynamically monitor tumors. However, compared with radial artery blood, the positive rate and CTC count were higher, suggesting the presence of central clearance [72]. According to the patient’s condition and the mode of treatment, blood samples from different sources can provide more meaningful information for the clinic. A prospective clinical study of 93 patients with early-stage NSCLC treated with stereotactic body radiation therapy showed that high levels of pretreatment CTCs (≥5 CTCs/ml) and persistent post-treatment CTCs were significantly associated with an increased risk of off-site recurrence [73]. This finding also suggests that CTC quantification has the potential to identify patients at higher risk of recurrence. Preliminary evidence indicates that the status and count of CTCs have potential for early diagnosis, staging and prognosis, which is of great significance for the timely adjustment and optimization of treatment [74, 75].

### Molecular characterization of CTCs

CTCs carry the molecular characteristics of primary tumor cells. A comprehensive analysis of the information shared by the CTC population can be used to judge the origin and diagnosis of tumors. CTCs also carry acquired mutations different from those found at the sites of exfoliated, which can be used to monitor tumor drug resistance and the acquisition of metastatic potential [76]. Combining these two types of information can provide unique insights on tumor heterogeneity at the level of the tumor genome, transcriptome, proteome and function [77, 78].

In recent years, immunotherapy has developed rapidly and brought new hope to patients previously treated with chemotherapy for refractory tumors. Immunotherapy mainly uses immune checkpoint inhibitors targeting programmed cell death protein-1/ programmed cell death-ligand 1 (PD-1/PD-L1). However, patients who can benefit from immunotherapy are limited. In addition, acquired drug resistance easily occurs during treatment. The molecular characteristics of CTCs can reveal cell-cell interactions and suggest available therapeutic targets, thus potentially allowing the identification of patients who can benefit from immunotherapy. The detection of CTC immune checkpoints (e.g., PD-L1 and CD47) can predict prognosis and treatment efficacy for patients receiving immunosuppressive drugs [79–81]. However, the predictive value varies with different tumor stages, suggesting that the innate immunity and acquired immune evasion mechanism of CTCs may be related to their metastatic potential. After preliminary verification in lung cancer, breast cancer and other cancers, CTCs were suggested to have the potential to become an independent prognostic biomarker of immunotherapy. There were both consistencies and differences in the expression of PD-L1 in tumor tissue and CTCs, and parallel evaluation of peripheral and local immunity provided more comprehensive information [82, 83]. In addition, the expression of the nuclear androgen receptor splice variant 7 (AR-V7) protein on CTCs is related to androgen receptor-targeted drug resistance, which can guide drug selection for metastatic castration-resistant prostate cancer (mCRPC) patients [84, 85]. The expression of specific proteins on CTCs could be used to predict drug sensitivity and select appropriate drugs.

The most appropriate targeted therapy regimen could be selected according to the genotypic changes of CTCs, and the efficacy could be evaluated over time. For example, the anaplastic lymphoma kinase (ALK) gene fusion mutation is a common strong carcinogenic driver gene in NSCLC, second only to EGFR. ALK inhibitors can effectively prolong the survival time of NSCLC patients with positive ALK gene fusion mutations and address the lack of drug availability after drug resistance. However, due to ALK rearrangement, patients will develop resistance to ALK inhibitors. Single CTC sequencing can be used to identify drug resistance mutations in patients with ALK rearrangement and evaluate the mechanism of heterogeneous drug resistance to facilitate clinical decision-making [86]. Breast cancer patients with HER2+ CTCs may benefit from corresponding targeted therapies [87].

With the development of next-generation sequencing (NGS) technology, the application of CTCs in high-throughput molecular diagnosis has become possible. Analyzing and comparing the consistency between gene mutations in CTCs and primary lesions/metastases [88, 89], combined with the clinical characteristics and treatment status of patients, will be helpful to understand the immune escape mechanism, drug resistance mechanism, and invasive ability acquisition mechanism [90] and find new signaling pathways.

## Clinical implications of CTCs for CRC

### Diagnosis and assessment of prognosis

Existing data have clearly demonstrated the clinical significance of CTCs in different stages of CRC. However, further investigations are needed to demonstrate whether CTCs can provide the expected value in clinical applications and guide clinical decision-making compared with tumor biomarkers that have been verified and widely accepted (Table 3).

**Table 3:** Clinical Application value of CTCs in CRC

Patient number	Technique	Marker	CTC cut-off	Patient ratio (%)	Significance	Ref
10	ClearCell FX LSC-MS	–	–	–	Differences in the metabolic profile can be used to discriminate CTCs andlymphocytes obtained from the same patient and CTCs of different cancer types.	[93]
50	RT-PCR	–	–	82.00	The CTCs number obtained by quantifying mRNAs of six CRC-related genes (CEA,EpCAM, CK19, MUC1, EGFR and C-Met) in the blood was associated with 2-year PFS.	[94]
132	CellSearch	EpCAM	≥3CTC/7.5 ml	19.00	Baseline and 4 weeks CTCs counts ≥3/7.5 mL in CRC patients with potentiallyresectable liver metastases were associated with decreased OS.	[95]
44	CellSearch	EpCAM	≥2CTC/7.5 ml	18.6	Preoperative CTCs counts ≥2/7.5 mL in CRC patients with resected liver metastaseswere associated with decreased DFS and OS.	[97]
130	MACS	CD45	≥2CTC/3.2 ml	51.54	Postoperative CTCs counts ≥2/3.2 mL in non-mCRC was associated with decreased RFS.	[99]
66	CanPatrol Multiplex mRNA-ISH	–	≥6CTC/5 ml	86.4	CTCs counts ≥6/5 ml was associated with decreased PFS and OS. LGR5 expressionin CTCs may serve as a marker for CRC metastasis.	[100]
78	MACS qRT-PCR	EpCAM CD45	–	–	Akt-2 (≥0.25 ng μl-1) expression in CTCs was associated decreased median PFS.	[103]
91	CanPatrol mRNA-ISH	–	≥3CTC/5 ml ≥1mCTC/5 ml	CTC 56.0 mCTC 50.5	Mesenchymal CTC counts ≥1/5 ml and COX-2 expression in mCTCs were associatedwith distance metastasis.	[104]
42	CellSearch	EpCAM	≥3CTC/7.5 ml	52.3	Patients with CTC ≥3/7.5 ml may benefit from the intensive 4-drug regimen (irinotecan,oxaliplatin, and tegafur-uracil with leucovorin and cetuximab).	[106]
55	MACS Flow Cytometry	EpCAM CD45	> 30CTC/ml	52.7	CTCs counts > 30/ml in mCRC patients treated with chemotherapy was associatedwith decreased PFS and OS.	[107]
138	ISET device-CTCBIOPSY	–	≥1CTC/2.5 ml	45.7	Post curative resection CTCs counts > 1/2.5 ml was associated shorter 3-year RFS rate.	[108]
589	CellSearch	EpCAM	≥3CTC/7.5 ml	41	Baseline CTCs counts ≥3/7.5 mL was associated with clinical or pathologic featuresassociated with poor Prognosis.	[110]
628	qRT-PCR	–	–	33.6	Plastin3 is a CTC marker specifically expressed in metastatic colorectal cancer cells.Preoperative PLS3-positive CTCs in peripheral blood were associated decreased DFSand OS.	[112]
14	ISET qPCR	–	–	60	The CTCs captured by the ISET system allows for counting, genotypic and phenotypiccharacterization. Using a qPCR method, KRAS exon 2 mutations were successfullydetected in CTCs of patients with RAS-WT mCRC who treated with first-linechemotherapy and monoclonal antibodies.	[116]
61	CellSearch ASONE Cell PickingSystem PCR/WGA	EpCAM	≥1CTC/7.5 ml	44.3	CTC heterozygosity and heterogeneity exist in KRAS status among CTCs within apatient and between CTCs and tumor tissues.	[117]
50	CellSearch Multiplex-PCR	EpCAM	≥3CTC/7.5 ml	46	CTCs counts ≥3/7.5 mL at baseline and day 21 after initiation of regorafenib wereassociated decreased PFS and OS. Patients had significantly increased EGFR expressionat day 21 and/or PD compared to baseline.	[118]
94	MACS RT-qPCR	EpCAM	–	–	Patients with higher gene panel expression (composed of GAPDH, VIL1, CLU, TIMP1,TLN1, LOXL3 and ZEB2) at baseline or increased gene panel expression duringtreatment had decreased PFS and OS. This CTC detection panel had increasedreliability compared to computed tomography scan.	[132]
106	MACS qRT-PCR	CD45 Pan-CK	≥5CTC/ml	100	HAI/target therapy with drugs selected by liquid biopsy precision oncotherapy isa safe and efficacious alternative therapeutic strategy for unresectable colorectalliver metastases patients.	[133]

**Fig. 3:**
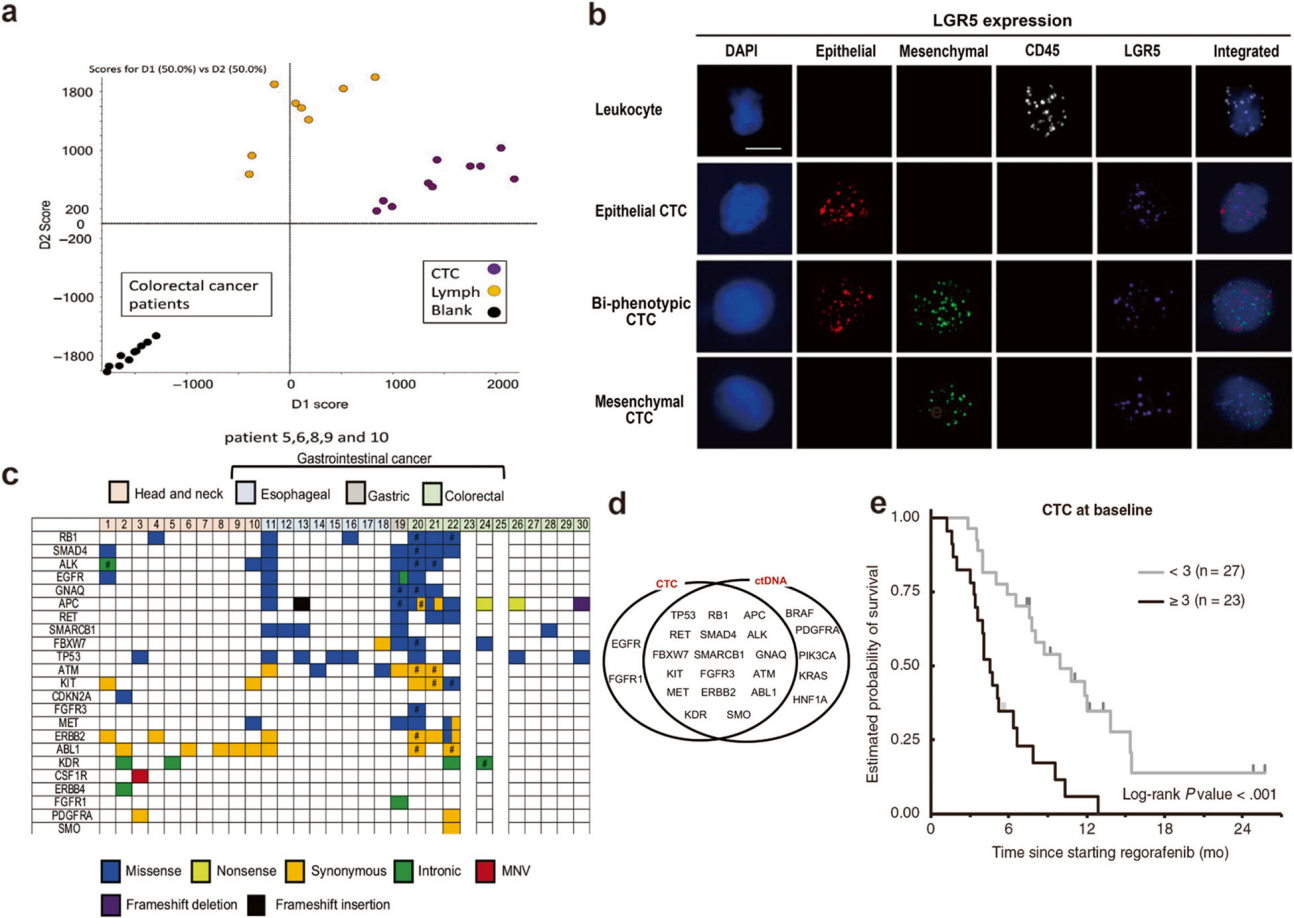
Molecular profiling and clinical application of CTCs. **a** The difference in the metabolic profile between CTCs and lymphocytes of CRC patients. The analysis of the metabolic profile of CTCs shows the potential for the early diagnosis and differential diagnosis of cancer; **b** Fish images of different CTC phenotypes; **c**, **d** The hot map of genomic alterations in CTC and ctDNA in head and neck cancer patients and CRC patients. The associations between genomic alterations detected in ctDNA and CTC in CRC; **e** Multivariable analysis of overall survival of mCRC patients treated with regorafenib, by CTC counts at baseline. **a** Copyright Wiley 2019. Reproduced with permission from reference [93]; **b** Copyright Springer Nature 2018. Reproduced with permission from reference [100]; **c**, **d** Copyright Wiley 2019. Reproduced with permission from reference [115]; **e** Copyright Wiley 2020. Reproduced with permission from reference [118]

The quantification of CTCs can be used for the early diagnosis of CRC. CTC counts differ significantly between patients with colorectal polyps and those with nonmetastatic CRC. These differences hold potential for disease screening and the diagnosis of suspected malignant lesions, including even indicating the anatomical location and differentiation of tumors [91]. A prospective clinical study presented at the 2018 American Society of Clinical Oncology (ASCO) conference used the CellMax biomimetic platform for early CRC screening [92]. A total of 620 samples (182 healthy controls, 111 patients with precancerous lesions, and 327 patients with stage I-IV CRC) were enrolled. The results of CTC detection were compared with the results of routine clinical diagnostic methods such as colonoscopy and biopsy, and the overall accuracy was 88%. The false positive rate reported in the healthy control group was low (3.3%), and the false negative rate of cancer patients was 16%, which was within the acceptable range. Yasmine et al. reported that the metabolic profiles of different types of cells are different. Living single-cell mass spectrometry (LSC-MS) can be used to distinguish lymphocytes and CTCs derived from the same patient, as well as CTCs from different cancer types [93] (Fig. 3a). They found that eicosanoids, acyl carnitine metabolites, and sterol lipids were specifically increased in CRC cells. The analysis of the metabolic profile of CTCs has the potential to enable the early diagnosis and differential diagnosis of cancer. Due to the heterogeneity of tumor cells, it is difficult to quantify CTCs accurately according to the biomarkers of the primary tumor. Li et al. quantified the CTCs of 50 CRC patients based on the mRNA levels of six genes (CEA, C-Met, MUC1, CK19, EGFR and EpCAM). The sensitivity of diagnosis was 87%, and the accuracy was 85% [94]. Compared with enteroscopy, CTC analysis is associated with better patient compliance and a lower economic burden. At present, colonoscopy and biopsy are still the gold standard for diagnosis, and further biopsies can be performed for CTC-positive patients. With further improvements in specificity, CTC detection technologies have the potential to completely replace tissue biopsy as a new diagnostic standard.

In addition to disease diagnosis, some studies have emphasized that CTC load can be used to predict recurrence and metastasis and evaluate patient prognosis. François et al. analyzed data obtained from the Unicancer Prodige-14 clinical study and found that for CRC patients with potentially resectable liver metastases, high CTC counts (≥ 3/7.5 ml) before treatment and 1 month after treatment were an independent prognostic factor for poor OS [95]. The CTC load before and after surgery also plays an important role in predicting recurrence and metastasis [96]. For CRC patients with resected liver metastases, even if the metastasis was completely resected, DFS and OS were often shorter when the preoperative CTC analysis was positive (positive threshold 2/7.5 ml) [97]. Postoperative CTC levels were significantly correlated with preoperative tumor biomarker CA125 levels and recurrence in patients with nonmetastatic CRC [98, 99]. The ratio of mesenchymal phenotype CTCs to total CTCs can be used to evaluate prognosis and metastasis [100] (Fig. 3b). Although the quantity of CTCs clearly has predictive value, counting alone is not sufficient to achieve a comprehensive understanding of the nature and characteristics of tumors. The molecular characteristics of CTCs can provide more accurate prognostic information. Metastasis is the main cause of death in CRC. It is a multistage and complex process, and only cells that meet specific conditions can continue to metastasize. The analysis of CRC tissue samples of different stages has revealed specific chromosomal variations corresponding to each metastatic stage [101]. The chromosome variation in CTCs are consistent with these tissue chromosome variation and corresponding metastasis stage, emphasizing that the molecular characteristics of CTCs has the potential to predict recurrence and metastasis [102]. For example, a mesenchymal phenotype is common in metastatic tumor cells, and patients with CTCs expressing the EMT-associated mRNA Akt-2 have worse prognosis [103]. A study detected the expression of cyclooxygenase-2 (COX-2) in CTCs with different phenotypes in 73 CRC patients. The expression of COX-2 in mesenchymal CTCs was closely related to metastasis [104], indicating the potential for early detection of metastasis and evaluation of prognosis. Nevertheless, the correlation between COX-2 expression in total CTCs and metastasis requires further research.

### Guiding precision medicine

#### Quantification

Patients with advanced CRC are mainly treated with multiline therapy, but the number of suitable patients is limited, and some patients have only one systematic treatment opportunity. Therefore, accurately selecting an effective therapeutic regimen that patients can tolerate is very important to improve prognosis. In addition, acquired drug resistance often occurs during treatment, and there is a certain delay in determining changes in lesions by medical imaging examination. Invasive tissue biopsy is not suitable for frequent use, which makes it difficult to adjust the treatment plan in a timely manner. Several studies have shown that the CTC count reflects tumor burden to some extent and increases with increasing tumor stage. Further investigations on the tumor TNM staging system showed positive correlations between the number detected CTCs and primary tumor size, depth of invasion, lymph node invasion, and distant metastasis, suggesting that CTCs are feasible markers for judging infiltration and metastasis [105]. Anti-EGFR therapy has been confirmed to improve outcome in wild-type RAS CRC. However, there are no recognized biomarkers or molecular markers to predict the prognostic value of systemic therapy. To find a biomarker that can identify patients who cannot benefit from the regimen before treatment begins, Matthew et al. detected CTCs in 42 patients with advanced CRC, and the data obtained from this study were modeled and compared with a larger sample of clinical data (CAIRO2). It was concluded that patients with a high baseline CTC level (≥3/7.5 ml) were more likely to benefit from intensive therapy [106]. Chou et al. also pointed out that the baseline number of CTCs before chemotherapy was an independent prognostic factor. A prognostic model was established by combining the baseline number of CTCs with other prognostic clinical parameters (past colorectal resection history, Eastern Cooperative Oncology Group (ECOG) score, tumor stage, and histological grade) to assist clinicians in identifying patients who could benefit from palliative treatment prior to the initiation of treatment [107]. Postoperative CTC positivity was identified as an independent prognostic risk factor for recurrence in nonmetastatic CRC patients undergoing radical resection, and 3-year relapse-free survival (RFS) was significantly lower in these patients than in postoperative CTC-negative patients [108]. This study illustrated that a postoperative CTC assessment was necessary to guide risk adjustment and individualized monitoring strategies. Compared with the main clinical tumor biomarkers of CRC (CEA, CA125...), changes in CTC count change are more sensitive and are usually positively correlated with disease progression defined by medical imaging examination. The joint application of these techniques can provide more accurate information and can be used to monitor the response to chemotherapy and disease progression/remission during treatment [109, 110].

#### Single-cell analysis

CTCs can represent aggressive tumor cell subsets as a whole, and the elimination of these cells is an important measure for preventing recurrence and metastasis. However, the overall assessment of all cells enriched from whole blood may be biased by the influence of lymphocytes. Therefore, precise analysis at single-cell resolution can provide a better understanding of cancer occurrence, development, therapeutic targets and drug resistance.

##### Whole-genome sequencing

CTCs carry the mutation characteristics of the primary tumor. However, the mutations of CTCs measured at different time points show heterogeneity with the mutations of the primary lesion, especially in patients with metastatic tumors. This inconsistency offers effective prognostic information [111]. Patients with metastatic CRC-specific genes expressed in CTCs are often prone to postoperative recurrence, and this information can be used to screen patients in need of postoperative adjuvant therapy. Plastin3 is specifically expressed in metastatic CRC. Preoperative plastin3-positive CTCs in peripheral blood are related to poor prognosis, especially in patients with Dukes B and Dukes C [112]. These patients should consider postoperative adjuvant chemotherapy. Genome sequencing of CTCs helps to reveal genetic differences between local and metastatic cells.

Currently, anti-EGFR therapy is approved for wild-type RAS CRC. With the emergence of EGFR-targeting drugs such as cetuximab and panizumab, the prognosis of CRC has improved [113]. However KRAS mutation leads to resistance to anti-EGFR therapy, and even patients with wild-type RAS may develop resistance to treatment due to a variety of mechanisms, resulting in a five-year survival rate of only 10 to 15% [114]. The mechanism of drug resistance may be revealed by analyzing CTC mutations and synthesizing the trend of their mutated gene expression levels over time [115] (Fig. 3c, d). CTCs reflect the real-time status of the tumor genotype, and the molecular profiles of CTCs can help guide treatment plans. Alexios et al. successfully detected KRAS exon 2 mutations in single CTCs from wild-type RAS CRC patients [116]. Yuurin et al. analyzed the mutations in codons 12 and 13 of the KRAS gene in single CTCs and corresponding tumor tissue samples from 7 CRC patients. They found heterogeneity and heterozygosity in KRAS status among the CTCs within a patient and between CTCs and tumor tissues [117]. Regorafenib is a vascular endothelial growth factor receptor (VEGFR) inhibitor, and Satoshi et al. found that the expression of EGFR in CTCs was significantly increased in patients who received regorafenib and were evaluated for progression after treatment compared to baseline CTCs. The authors concluded that increased EGFR expression may be a pattern of resistance and provided supporting evidence for the synergistic effect of regorafenib and anti-EGFR drugs [118] (Fig. 3e). Human epidermal growth factor receptor 2 (HER2) is a member of the EGFR family. Studies have shown that anti-EGFR therapy has poor efficacy in patients with HER2-amplified metastatic CRC, indicating that HER2 amplification can be used as a prognostic biomarker. HER2 is also a potential therapeutic target in CRC. Several clinical studies have shown that anti-HER2 dual-target combination therapy significantly improves the prognosis of HER2-positive CRC patients, with an objective response rate reaching 30% [119, 120].

CTCs carry both primary tumor mutations and acquired mutations that differ from the primary tumor. Kong et al. collected preoperative peripheral blood samples and corresponding primary tumor tissue samples from 48 CRC patients and sequenced a gene panel including 39 drug therapy targets and frequently mutated genes. The CTCs were found to carry mutational characteristics similar to those of the primary tumors, and several treatment-related gene mutations (APC, KRAS, TP53, ERBB3, FBXW7 and ERBB2) were detected. They pointed out that the sequencing of single CTCs obtained before surgery and identification of mutations related to targeted therapy may provide information for more timely and accurate treatment interventions [111]. Other emerging therapeutic targets for CRC, including the expression of NrF2/Keap1, NRG1, GARP, are also related to the treatment and prognosis of CRC patients [121–123]. The detection and analysis of drug therapy targets on CTCs is of great significance for the development of relevant clinical research.

Immunotherapy is of great significance for patients with advanced refractory CRC, and the main obstacle is how to accurately select the suitable population. PD-L1 expression is an effective indicator for screening lung cancer and melanoma patients who could benefit from immunotherapy; however, the value in CRC is limited. Currently, only CRC patients with microsatellite instability high (MSI-H) can benefit from immunotherapy alone. Approximately 95% of patients with Lynch syndrome and genetic CRC have tumor microsatellite instability (MSI). This type of tumor has higher immunogenicity and a large number of activated tumor-infiltrating lymphocytes. However only 5 to 8% of patients have MSI-H tumors. In addition, the tumor mutation burden (TMB) and comprehensive positive score (CPS) of PD-L1 can only provide a certain reference value. POLE and POLD gene mutations are more promising indicators for microsatellite stable (MSS) CRC [124]. Stenzinger et al. detected somatic POLE mutations in up to 12.3% of MSS CRC patients [125]. In addition, for MSS patients, those with POLE/POLD mutations showed high TMB and higher PD-L1/PD-1 gene expression levels. Overall, the detection of POLE/POLD mutations is meaningful in guiding immunotherapy for CRC, especially for MSS CRC patients [126–128].

##### Transcriptome analysis

Transcriptome analysis has been widely used to detect gene expression levels (mRNA) in tumor cell populations to understand the heterogeneity between tumors. Classifying tumors into subgroups using different molecules can help researchers predict the treatment response and prognosis of patients. However, transcriptome analysis of cell populations cannot reveal intratumoral heterogeneity. Therefore, to comprehensively understand intratumoral and intertumoral heterogeneity, transcriptome analysis at single-cell resolution has attracted extensive attention. The advantages of real-time detection and the better response to heterogeneity make CTCs a suitable object for single-cell transcriptome analysis [129].

Cells expressing leucine-rich repeat-containing g-protein coupled receptor 5 (LGR5) represent the origin of intestinal epithelial tumors. Increased LGR5 expression is closely related to the occurrence and development of a variety of cancers, including CRC, and is associated with poor prognosis [130, 131]. Wang et al. enriched the CTCs of 57 CRC patients and evaluated the expression level of LGR5 in each CTC by a multiple-mRNA in situ hybridization (ISH) assay. They concluded that LGR5 expression in CTCs can be used as an indicator to judge metastasis [100]. Yolanda et al. designed a multigene expression panel including GAPDH, VIL1, CLU, TIMP1, TLN1, LOXL3 and ZEB2. CTCs in peripheral blood were captured, and mRNA was extracted from 94 CRC patients. Quantitative real-time PCR was used to quantify mRNA and reflect the expression levels of the abovementioned genes. Prognosis was worse if the expression level of the above genes increased during treatment. After one cycle of treatment (4 weeks), patients who cannot benefit from the current regimen could be identified. Compared with computed tomography (CT), evaluation of the treatment response using CTCs is more sensitive and reliable [132]. It is possible to change an ineffective treatment plan as soon as possible, save time spent evaluating the treatment response, and reverse patient outcomes in a timely manner. Li et al. constructed a multiple gene-based algorithm named the six-gene assay, which consists of six CRC-related genes (CEA, EpCAM, CK19, MUC1, EGFR, and C-Met). CTC quantification was performed for blood samples of 50 patients with recurrent CRC by detecting the mRNA expression levels of the above genes [94]. Compared with the CEA assay, the six-gene assay was more sensitive and accurate in diagnosis and in predicting the 2-year DFS rate. Regorafenib is a multitarget anticancer drug that can effectively improve the survival rate of advanced CRC. However, some patients cannot benefit from it, and the mechanism of primary and acquired drug resistance remains to be studied. Matsusaka et al. performed a clinical study of 50 CRC patients with advanced multiple metastases who were tested for CTCs at baseline, 21 days after the initiation of regorafenib, and after progression. The mRNA levels of EGFR in CTCs were quantified, indicating that the EGFR level at the time of progression was significantly increased compared with that at baseline, suggesting upregulation of EGFR in CTCs may be a mechanism of acquired drug resistance under regorafenib therapy [118].

In addition to single-cell analysis, CTCs can be harvested by culture and then tested for drug sensitivity to select the appropriate drug. One study compared the outcomes of patients who were treated based on a CTC drug sensitivity test (44 patients) with those who were treated based on the traditional method (62 patients). The authors found that the former had significantly longer PFS at 5 months and no drug-related adverse events [133], indicating that CTC drug sensitivity testing is a safe and effective strategy. While a median PFS extension was observed, it did not reach a significant level, and larger cohort studies are needed to demonstrate the potential of this approach.

## Conclusion and future development directions

Decades of research have led to significant advancements in the clinical utility of CTCs in CRC patients. CTCs counting is already FDA-approved for disease monitoring and prognosis assessment in CRC. As a form of liquid biopsy, CTCs also hold great value in facilitating the implementation of precision medicine in CRC. Analyzing CTCs could offer a unique minimally invasive approach to early diagnosis and the characterization and monitoring of dynamic changes in tumor heterogeneity at the genomic, transcriptomic, proteomic and functional levels. However, significant challenges remain before CTCs can be adopted for widespread clinical use in CRC. There is a need to develop and verify detection methods that can achieve high sensitivity and specificity. Among them, nanotechnology is the most active area of research. In addition to technological challenges, thresholds and standards vary between studies, making it difficult to perform a comparative analysis. Therefore, unified standards at different stages are needed. The comprehensive analysis of CTCs and the clinical characteristics of patients will be a powerful tool to promote the development of precision medicine.

## Data Availability

Not applicable.

## Abbreviations

CRC: Colorectal cancer
CTC: Circulating tumor cell
ctDNA: Circulating tumor DNA
miRNA: microRNA
EVs: Extracellular vesicles
EMT: Epithelial-mesenchymal transition
MET: Mesenchymal-epithelial transition
EpCAM: Epithelial cell adhesion molecule
FDA: Food and Drug Administration
DAPI: 4′,6-diamidino-2-phenylindole
QDs: Quantum dots
PEO: Polyethylene oxide
FRs: Folate receptors
FA: Folic acid
MACS: Magnetic-activated cell sorting
rVAR2: Recombinant VAR2CSA
STn: Sialyl-Tn
LC: Lung cancer
NSCLC: Non-small cell lung cancer
RCC: Renal cell carcinoma
EGFR: Epidermal growth factor receptor
ECM: Extracellular matrix
AuNPs: Gold nanoparticles
MCF-7: Michigan Cancer Foundation-7
PSA: Prostate-specific antigen
OS: Overall survival
LR: Likelihood ratio
DFS: Disease-free survival
DDFS: Distant disease-free survival
BC: Breast cancer
MBC: Metastatic breast cancer
HR: Hormone receptor
Let: Letrozole
Bev: Bevacizumab
PD-1: Programmed cell death protein-1
PD-L1: Programmed cell death-ligand 1
AR-V7: Androgen receptor splice variant 7
mCRPC: Metastatic castration-resistant prostate cancer
ALK: Anaplastic lymphoma kinase
NGS: Next-generation sequencing
ASCO: American Society of Clinical Oncology
LSC-MS: Living single-cell mass spectrometry
COX2: Cyclooxygenase-2
ECOG: Eastern Cooperative Oncology Group
FRS: Relapse-free survival
VEGFR: Vascular endothelial growth factor receptor
HER2: Human epidermal growth factor receptor 2
MSI-H: Microsatellite instability high
TMB: Tumor mutation burden
MSS: Microsatellite stable
LGR5: Leucine-rich repeat-containing g-protein coupled receptor 5
ISH: In situ hybridization
CT: Computed tomography
ofCS: Oncofetal chondroitin sulfate
ALL: Acute lymphoblastic leukemia
HCC: Hepatocellular Carcinoma
PAAD: Pancreatic adenocarcinoma
PC: Prostate cancer
GBMLGG: Glioma
mCTC: Mesenchymal CTCs
PCR: Polymerase Chain Reaction
HAI: Hepatic artery infusion
